# DOES AEROBIC EXERCISE AFFECT MEMORY, ATTENTION, WORKING MEMORY, AND FATIGUE AFTER ACQUIRED BRAIN INJURY? A SINGLE-BLINDED, RANDOMIZED CONTROLLED PILOT STUDY

**DOI:** 10.2340/jrm.v58.44745

**Published:** 2026-04-16

**Authors:** Eygló INGÓLFSDÓTTIR, Ulla BERGFELDT, Märta Berthold LINDSTEDT, Lennart BERGFELDT, Tie-Qiang LI, Tomas JONSSON, Marika C. MÖLLER

**Affiliations:** 1 Department of Rehabilitation Medicine, Danderyd University Hospital, Stockholm; 2 Department of Molecular and Clinical Medicine, Institute of Medicine, Sahlgrenska Academy, University of Gothenburg, Gothenburg; 3 Department of Medical Physics, Karolinska University Hospital, Huddinge; 4 Department of Clinical Science, Division of Rehabilitation Medicine, Danderyd Hospital, Stockholm, Sweden

**Keywords:** stroke, traumatic brain injury, cognition, fatigue, aerobic exercise, magnetic resonance imaging, neuronal plasticity

## Abstract

**Objective:**

To explore potential effects of aerobic exercise on cognitive function, fatigue, and neuroplasticity after stroke or traumatic brain injury.

**Design:**

A randomized controlled pilot study.

**Subjects/Patients:**

Twelve participants, 10 men and 2 women aged 16–65 years diagnosed with acquired brain injury (moderate to severe stroke or traumatic brain injury), enrolled in an outpatient rehabilitation programme at least 3 months post-injury.

**Methods:**

The intervention group (*n* = 6) participated in 30 min of aerobic exercise 3–4 times/week for 8 weeks during their outpatient rehabilitation. The control group (*n* = 6) received routine physical therapy. In addition, both groups received rehabilitation according to their rehabilitation plan. Neuropsychological and endurance testing was performed before and after the intervention and at a 3-month follow-up. Functional magnetic resonance imaging was performed before and after the intervention.

**Results:**

Preliminary findings show no significant difference between the groups regarding cognitive variables, fatigue, or neuroplasticity. However, working memory/executive demanding processing speed, measured with the Paced Auditory Serial Addition Test, improved significantly (*p* = 0.042) in the aerobic exercise group and there were trends of improvements on several other executive tests in the aerobic exercise group. Automatic visual search speed improved in the control group (*p* = 0.027).

**Conclusion:**

The question of whether aerobic exercise promotes cognition and fatigue after brain injury remains unanswered. This randomized controlled pilot study and its preliminary findings indicate greater improvements in executive processing speed in the aerobic exercise group, while more automatized attention speed improved more in the control group. The experiences from this study might facilitate the design of future studies on this intriguing topic.

**Trial registration:**

ClinicalTrials.gov Identifier: NCT07429526

## LAY ABSTRACT

Acquired brain injury can affect cognition and cause fatigue. Aerobic exercise has been linked to improved cognition in the general population. This is thought to occur through boosting neuroplasticity, that is to say the brain’s ability to change and adapt. We explored the potential effects of aerobic exercise after acquired brain injury and investigated whether any changes in the brain could be detected. This was done through an 8-week period of 30 min of aerobic exercise, 3–4 times a week, for a group of 6 patients compared with 6 patients who did their usual training. Functional magnetic resonance imaging was used to detect any potential changes in the brain. The preliminary results showed no significant benefits for the treatment group. However, compared with before treatment, the treatment group showed preliminary improvements in how quickly they could process complex information. The control group showed preliminary improvements in simple visual scanning. Larger studies are needed.

Acquired brain injuries (ABI) such as stroke and traumatic brain injury (TBI) often lead to fatigue and cognitive deficits such as impaired attention and memory. This can make it difficult to plan and organize activities of daily living as well as making it harder to return to work and participate in social activities (1). Consequently, cognition is strongly associated with quality of life after stroke and TBI (2, 3). A focus on improved cognitive functioning is therefore an important part of brain injury rehabilitation, with the goal of optimal recovery.

Aerobic exercise (AE) has been linked to improvement in several aspects of cognition in the general population (4–7). Theories behind the improvements have primarily focused on vascular changes, increased grey matter volume, changes in cerebral blood flow, longer dendrites with a more complex morphology, alterations in neurotransmitter systems, increased angiogenesis, higher levels of synaptogenesis, higher levels of neurogenesis, and increases in growth factors such as brain-derived neurotrophic factor (BDNF) (8–16). Any exercise or treatment that boosts neuroplasticity is of interest in brain injury rehabilitation as it might optimize the outcome. However, whether training-induced neuroplasticity may influence cognition and fatigue after an ABI has still not been determined. As pointed out by Montero-Almagro et al., the injury due to an ABI may influence the physiological response in the brain, specifically the injured area, thus not giving the same response as in healthy individuals (17).

Clinical practice guidelines for rehabilitation after stroke or TBI do not include AE as a way of influencing cognition (18, 19), or fatigue (20). This also applies to the national guidelines for stroke care in Sweden (21), where this study was performed. This pilot randomized controlled trial (RCT) was designed to explore the effects of aerobic exercise on attention, memory, working memory, and fatigue during rehabilitation of stroke and TBI patients. Furthermore, fMRI was included in the study to explore any changes in neuroplasticity that could be explained by the intervention.

## METHODS

### Study design

This was a pilot RCT with a parallel group design where the intervention consisted of 30 min of aerobic exercise, 3–4 times/week for 8 weeks, included in a rehabilitation programme based on current guidelines and patients’ individual needs. The control group received rehabilitation according to their rehabilitation programme, not including aerobic training. The patients had suffered from either TBI or stroke. Severity of injury was classified using the Glasgow Outcome Scale – Extended (GOSE) (22). Patients were eligible for rehabilitation at the clinic if they scored 6 or lower on the assessment scale. The median score among patients admitted was 5. Admission required a verified brain injury confirmed by magnetic resonance imaging (MRI) or computed tomography (CT). Only data from baseline and from the 8‑week follow‑up are presented in this study.

### Setting

The study took place in an outpatient brain injury rehabilitation setting. All patients were currently in a rehabilitation programme with a frequency of at least 3 days/week divided into 3 parts: (*i*) functional training aiming at improving physical, cognitive, and communicational skills; (*ii*) learning strategies for coping with functional disabilities; and (*iii*) preparation for returning to work when that was regarded as possible. Rehabilitation included both individual and/or group sessions with the rehabilitation team (occupational therapist, speech therapist, social worker, psychologist, physical therapist, a nurse, and a physician) and with some computerized training. Enrolment took place between 2013 and 2022.

### Participants and study procedure

After a 2-week evaluation period, at least 3 months after the stroke or TBI, the test results were reported during a team meeting. Patients were considered eligible for enrolment if they demonstrated performance of at least 1 standard deviation (SD) below normal in at least 1 of the cognitive tests for verbal memory, visual memory, working memory, or attention. The study coordinator, who was also a team member, would then proceed and check whether the patient fulfilled all other inclusion criteria and none of the exclusion criteria. If so, the patient was a candidate for the study. For enrolment criteria, see Table I. The only exclusion criterion that had to do with physical sequelae after ABI was the inability to reach 60–80% of the estimated maximal heart rate (HRmax = 220 − age) during aerobic testing and training. This, however, allowed patients with a wide range of physical sequelae due to their injury the opportunity to participate. The range went from hemiplegic but ambulatory with a crutch short distance, to having no physical neurological symptoms after the injury.

**Table I:** Enrolment criteria

Inclusion criteria	Exclusion criteria
– First stroke/TBI with CT/MRI verified injury, (moderate to severe brain injury) – Age 18–65 years – At least 3 months after injury – Fluent in Swedish – Selection criteria Cognition: – at least 1 standard deviation (SD) below age-adjusted normal in at least 1 of the following tests: verbal memory, visual memory, working memory, or attention – able to follow instructions during neuropsychological (NP) testing. – Able to reach 60–80% of estimated maximal heart rate (HRmax = 220 – age) during aerobic testing and training	– Severe heart disease – Cognitive defects, not due to the actual stroke or TBI – Moderate depression/anxiety, defined as >10 respectively on HADS – Severe aphasia – Not fulfilling criteria for MRI (such as pregnancy, having metal parts implanted in the body, afraid of cramped spaces)

Patients who were considered candidates received written information concerning the study from the study coordinator and were asked if they were interested in participating. Those willing gave written consent and were thereafter randomized into 2 different groups, the intervention group, or the control group (CG). All assessments were performed before starting the study, after 8 weeks of participation and at a 3-month follow-up. The fMRI evaluation was performed before and after 8 weeks of participation.

**Fig. 1:**
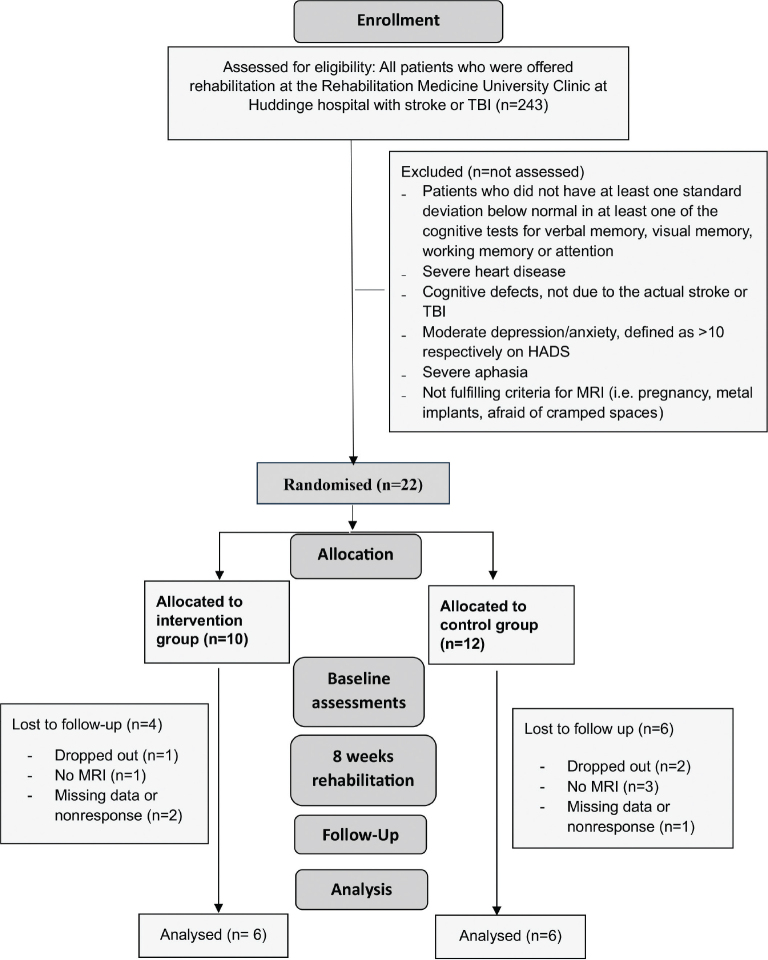
Consort flowchart.

The randomization was done by having the participant draw from a series of numbers with an equal amount of even and uneven numbers. The even numbers would place the patient in the AE group, the uneven in the CG. The patients could not see the numbers they picked and were thus blinded to group allocation. All team members except for the physical therapist (PT) responsible for the participant’s physical therapy, were also blinded to the group allocation. Before the intervention started, all patients underwent an fMRI investigation. Fig. 1 shows a Consort flowchart.

### Intervention

All patients received rehabilitation according to an individualized rehabilitation plan. Those who were randomized into the intervention group received 30 min of AE, 3–4 times/week for 8 weeks during their training with their physical therapist, as well as rehabilitation according to their rehabilitation plan. The AE training was performed on a stationary bike with an intensity of 60–80% of each participant’s estimated maximum heart rate (HR) (HR max = 220 – age). The exercise sessions were supervised by the treating physical therapist. During the exercise session, an HR monitor was strapped around the chest and the actual HR displayed on a monitor in front of the patient together with a note showing the target HR interval. Perceived exertion was evaluated using the Borg RPE scale (23). If needed, the therapist guided the patient on following the target HR interval. The patients in the control group received physical therapy according to current guidelines.

### Data sources/measurements

The following tests and questionnaires were used at baseline, after 8 weeks of rehabilitation, and at a 3-month follow-up. Detailed information on the instruments is presented in Table II.

**Table II:** Detailed information on the tests and questionnaires used at baseline, after 8 weeks of rehabilitation, and at 3-month follow-up

Tests	Description
**Neuropsychological tests**	
Delis–Kaplan executive function system (D-KEFS) Color Word Test	Measures the ability to inhibit automized verbal responses and the ability to use cognitive shifting (24). The test has 4 conditions: (*i*) *naming* the colours of squares (red, blue, or green, (*ii*) *reading* colour words printed in black, (*iii*) naming the printed colour of the colour words red, blue, or green printed in a different colour, which means inhibition of an over-learned function of reading the word; (*iv*) repeatedly *switching* between (a) naming the printed colour of the colour words red, blue, or green and (b) reading the words as quickly as possible. In this condition the person needs to keep track of clues that indicate rule change. Sub tests 1 and 2 are contrast tests and used for comparison between the sub-tests 3 and 4, i.e., the more complex tasks. The faster the time, the better which means that lower values represent better result
Rey Auditory Verbal Learning Test (RAVLT)	A test of verbal learning and long-term memory (25). The RAVL Test consists of a 15 noun-word list (list A) that is read to the participants. After the presentation of list A, the participant is requested to recall as many words as possible. The procedure is repeated 5 times, and after each trial recall is recorded and all 5e recall trials are summed into one score (= immediate recall score). After 5 presentations of list A, an interference list of 15 other nouns (list B) is read, and the participants are asked to recall as many words as possible. Immediately after the recall of list B, the participants are again asked to recall list A. Delayed recall of list A is measured 30 min after the immediate recall (= delayed recall score). The higher the value the better the result
Wechsler Adult Intelligence Scale (WAIS)- III Coding	Measures psychomotor processing speed, attention, and working memory. The task demands the participant to pair symbols with numbers for 120 s. The score equals the number of symbols correctly recorded. The test was presented according to the manual. The higher the scores the better the results (26)
WAIS-IV Digit Span	Measures verbal attention span and working memory. The participant is asked to repeat a series of digits in the same (attention span) or reverse order (working memory). The length starts with 2 digits and ends with 9 digits, 2 trials per length. After 2 consecutive errors at the same span length the test is aborted. The test was presented according to the manual. The higher the value the better the results (27)
Paced Auditory Serial Addition Test (PASAT)	Measures auditive processing speed, attention, and working memory (28). The test consists of 60 digits from 1–9 presented at a standardized pace of 2.4 s between each of the digits. The task is to sum each digit with the previously presented one before the next digit is presented. The test was presented according to the manual. The maximum score is 60. This test was also used to measure cognitive fatigability as declining performance in terms of an increased number of incorrect responses on the PASAT. The responses were divided into 5 sections of 12 numbers each, where the number of correct answers in the first section was subtracted from the number of correct answers in the last section. Fatigability was defined as a lower score at the end of the test compared with the beginning, giving a negative value (29)
Ruff 2 & 7 Selective Attention Test (Ruff 2 & 7)	Measures visual automatic detection speed and accuracy, and controlled search speed and accuracy (8). The test consists of 20, 15-s trials, where the subject must identify and cancel the target digits 2 and 7 among distractors. The distractors consist of letters (automatic selective attention) and other digits (controlled selective attention) for 10 trials each. Speed of attention is measured as the total number of correctly identified targets. The accuracy calculated as the total number of correct targets divided by the (total number of correct targets + total number of errors) multiplied by 100 according to the manual. A higher score equals better performance (30)
**Test of endurance and exertion**	
Åstrand sub-maximal endurance test	Performed on a Monark stationary bike connected to a digital test program (Monark Test Software 839E/939E) (31)
Borg Rating of Perceived Exertion (RPE)	Measures the level of exertion (23)
**Questionnaires**	
Hospital Anxiety and Depression Scale (HADS)	Measures depression and anxiety. The HADS consists of 2 subcategories: anxiety and depression. Both subscales range from 0 to 21, where scores between 8 and 10 indicate possible presence of anxiety or depression, while a score >10 indicates a higher likelihood of this (32)
Mental Fatigue Scale (MFS)	MFS is a fatigue scale, consisting of 15 questions rated on a 7-point scale based on intensity, frequency, and duration of symptoms (33). The scale has a total score of 44, and 10.5 is the cut-off score for mental fatigue (34)

Neuropsychological tests:

- Delis–Kaplan Executive Function System (D-KEFS) Color Word Interference Test (CWIT) (24).
- Rey Auditory Verbal Learning Test (RAVLT) (25).
- Wechsler Adult Intelligence Scale (WAIS) – III Coding (26).
- WAIS-IV Digit Span (27).
- Paced Auditory Serial Addition Test (PASAT) (28). Fatigability was defined as a lower score at the end of the test compared with the beginning, giving a negative value (29).
- Ruff 2 & 7 Selective Attention Test (Ruff 2 & 7) (30).

Tests of endurance and exertion:

- Åstrand sub-maximal endurance test (31) was performed on a Monark stationary bike connected to a digital test program (Monark Test Software 839E/939E).
- The Borg Rating of Perceived Exertion (RPE) (23).

Questionnaires:

- Hospital Anxiety and Depression Scale (HADS) (32).
- Mental Fatigue Scale (MFS) (33, 34).

### Neuroimaging

Subjects underwent 2 fMRI exams before and after an 8-week intervention using a 3T Siemens Magnetom Prisma scanner (Siemens Healthineers, Erlangen, Germany) with a 64-channel head coil. The protocol included 3D MRI scans: T1-weighted imaging (T1W), T2-weighted imaging (T2W), T2 fluid attenuated inversion recovery (T2-FLAIR), diffusion tensor imaging (DTI), perfusion mapping with pseudo-continuous arterial spin labelling (PCASL), and an 8-min resting-state fMRI session. The fMRI used a blood oxygen level dependent (BOLD) sequence (TE/TR = 33/2500 ms, flip angle = 90°, 40 oblique axial slices, FOV = 230 x 230 mm, 64 x 64 matrix, GRAPPA acceleration).

Functional connectivity (FC) analysis was performed to examine brain region interactions during rest, using methods to remove movement-related artefacts. The voxel size was 4 x 4 x 4 mm³, with bicubic interpolation and Gaussian in-plane blurring. Preprocessing was done with AFNI software (35) followed by FC analysis using the quantitative data-driven analysis (QDA) framework to identify functional hubs. Pearson’s cross-correlation coefficients were calculated for each voxel, resulting in 2 FC metrics: connectivity strength index (CSI) and connectivity density index (CDI). CSI measures local connectivity strength, while CDI measures local connectivity density. Patient FC metrics were compared with a cohort of 227 adults aged 18–76, as reported by Li et al. (36).

### Statistical methods

*Methods used for neuropsychological testing and fatigue.* Median (range) was used for descriptive purposes. Non-parametric tests were used to obtain as robust results as possible. Mann–Whitney *U* test and Fisher’s exact test were used for between-group comparisons and Wilcoxon’s test for within-group comparisons. To examine individual differences between baseline and 8 weeks of training, a *d*-value was calculated by subtracting the result from baseline from the result from the measurement at 8 weeks; *p* < 0.05 was considered statically significant. Due to the small sample size and the exploratory nature of this study, we did not compensate for multiple testing.

*Group-level statistics of fMRI data.* The experimental results were analysed at the group level using a multivariate approach. To investigate the influence of aerobic exercise on FC metrics (CSI and CDI), a voxel-wise 3-way ANOVA was conducted using the AFNI program (3dANOVA3; https://afni.nimh.nih.gov/) with option-type 5. The primary factors included the intervention effect (control vs aerobic exercise) and repeated measurements over time. Subjects were considered a random factor, nested within the intervention effect, due to different subject groups. The ANOVA model included terms for main effects and interactions, all of which were evaluated using hypothesis tests (*F*-tests). Statistical significance was assessed using a voxel-wise *p*-threshold of *p*<0.01 without multiple comparison correction. To enhance the statistical significance, a minimum voxel cluster size of 15 contiguous voxels was enforced.

### Association between FC metrics and cognitive performance

To explore relationships between FC metrics, cognitive performance, and demographic characteristics, we conducted regression analyses between FC estimates and cognitive performance metrics in a data-driven manner. For dimensionality reduction and to capture the underlying structure of clinical data, a principal component analysis (PCA) was initially performed. The top 2 PCA components were then used as predictors in voxel-wise regression analyses with FC metrics as the dependent variable. The statistical significance of the regression analyses was assessed using a two-step approach. First, a voxel-wise threshold of uncorrected *p*<0.01 (*t*-score ≥2.85) was applied to identify initial cluster candidates. Subsequently, a minimum voxel cluster size of 18 contiguous voxels was enforced to enhance the statistical significance. Using the detected region of interest (ROI) as masks, mean values for the FC metrics were calculated, providing a more focused analysis of the relationship between FC and cognitive performance within specific brain regions.

## RESULTS

In this study only baseline data and those after 8 weeks of participation are reported.

### Participants

Twenty-two patients started in the study. Three dropped out, 1 from the AE group and 2 from the CG before ending the 8-week period. Three had missing data or non-response. Four (1 from the AE group and 3 from the control group) of the remaining 16 patients had to be excluded from the fMRI part because of a technical problem at the MRI unit. Furthermore, recruitment of patients was limited during the COVID pandemic due to hospital restrictions.

The reasons for terminating participation varied. There were patients in the AE group who found the training too hard, and those in the control group who found their physical therapy sessions too easy. There were also cases of participants discharging themselves from rehabilitation and cases of participants being hospitalized for medical reasons.

Twelve patients with ABI, 10 men and 2 women, concluded all parts of the study. The mean age was 52 (range 26–64) years. They participated in the study on average 6.3 (range 3–18) months after injury. Seven patients had a diagnosis of cerebral infarction, unspecified, 3 intracerebral haemorrhage unspecified, 1 unspecified focal TBI, and 1 subarachnoid haemorrhage. For patient characteristics, type of injury according to ICD-10, other medical diagnoses and medications of importance see Table III. The 2 groups were well balanced with regard to all baseline variables and test results.

**Table III:** Patient characteristics. Mann–Whitney U test and Fisher’s exact test for comparison between groups

Factor	Total (*n* = 12)	Intervention group (*n* = 6)	Control group (*n* = 6)	Group diff.
Age mean (SD; range)	52.3 (10.5; 26–64)	54.3 (14.4; 26–64)	50.3 (5.0; 41–55)	*p* = 0.132
Gender, men, *n* (%)	10 (83%)	6 (100%)	4 (67%)	*p* = 0.455
Education years mean (SD; range)	12.6 (1.4; 11–15)**	12.6 (1.3; 12–15)*	12.6 (1.5; 11–15)	*p* = 1.000
Type of injury	Stroke, *n* = 11 TBI, *n* = 1	Stroke, *n* = 5 TBI, *n* = 1	Stroke, *n* = 6	N/A
Months from injury to inclusion, mean (SD; range)	6.3 (4.5; 2–18)	5.2 (2.3; 2–8)	7.5 (6.0; 2–18)	*p* = 0.589
HADS – depression median (range)	2 (0–9)	2 (1–4)	2.5 (0–9)	*p* = 0.818
HADS – anxiety median (range)	4 (1–7)	3.5 (1–7)	4.5 (2–7)	*p* = 0.485
ICD 10 diagnosis	I63.9 (*n* = 7) I61.9 (*n* = 3) S06.30 (*n* = 1) I60.9 (*n* = 1)	I63.9 (*n* = 4) I61.9 (*n* = 1) S06.30 (*n* = 1)	I63.9 (*n* = 3) I61.9 (*n* = 2) I60.9 (*n* = 1)	
Other medical diagnosis Hypertension Epilepsy	(*n* = 7) (*n* = 1)	(*n* = 3) (*n* = 0)	(*n* = 4) (*n* = 1)	
Medication described after admission	AD (*n* = 1)	AD (*n* = 1)	AD (*n* = 0)	
Taking beta blockers on admission	(*n* = 3)	(*n* = 1)	(*n* = 2)	
Taking AD on admission	(*n* = 3)	(*n* = 1)	(*n* = 2)	

* 1 missing;

** 2 missing.

HADS: Hospital Anxiety and Depression Scale; I63.9: Cerebral infarction, unspecified; I61.9: Intracerebral haemorrhage, unspecified; S06.30: Unspecified focal TBI; I60.9: Subarachnoid haemorrhage; AD: antidepressants.

### Effects of treatment: baseline vs 8 weeks of treatment

Cognitive functioning at baseline and after 8 weeks of treatment is presented in Table IV. There were no significant differences between the groups either at baseline or after 8 weeks of treatment regarding cognitive variables and fatigue. We found no between-group differences regarding the change from baseline to after 8 weeks of treatment; however, there was a trend of better mental flexibility (CWIT Switching) in the aerobic exercise group (*p* = 0.093), see Table V. We also found some significant intragroup differences from baseline to 8 weeks of treatment regarding some of the cognitive variables. In the aerobic exercise group, working memory/executive demanding processing speed measured with PASAT test improved significantly (*p* = 0.042) and there were trends of improvements on several other executive tests. In the control group, automatic visual search speed improved (*p* = 0.027). For details see Table VI. This is an exploratory RCT pilot study involving a selected cohort of patients with ABI and the results should therefore be looked at as preliminary effects.

**Table IV:** Cognitive functions and fatigue at baseline (B) data and after 8 weeks of treatment (A, in bold font). Standard scores (ss) or *T*-scores (*T*-s) are presented for most of the variables. Mann–Whitney test was used for comparison between groups

Factor	Total (*n* = 12)	Intervention group (*n* = 6)	Control group (*n* = 6)	Group diff.
Executive functions				
• CWIT Inhibition (ss) B	9.5 (1–14)	8.5 (1–14)	10 (6–14)	0.699
• *CWIT Inhibition (ss) A*	**10.5 (2–14)**	**11 (2–13)**	**10 (8–14)**	**1.000**
• CWIT Switching (ss) B	8.5 (1–14)	8 (1–14)	8.5 (2–12)	0.818
• *CWIT Switching (ss) A*	**9.5 (2–14)**	**10 (4–14)**	**9 (2–14)**	**0.485**
Speed				
• CWIT Naming (ss) B	6 (1–12)	5.5 (1–8)	7.5 (6–12)	0.093
• *CWIT Naming (ss) A*	**9 (1–12)**	**9 (1–11)**	**8.5 (7–12)**	**0.818**
• CWIT Reading (ss) B	7 (1–10)	7 (1–10)	6.5 (1–10)	0.818
• *CWIT Reading (ss) A*	**8 (6–11)**	**6.5 (6–11)**	**8.5 (6–11)**	**0.310**
Attention				
• PASAT (raw scores) B	39 (17–55)	41 (24–55)	39 (17–49)	0.394
• PASAT (raw scores) A	44 (13–60)*	53 (29–60)*	**42.5 (13–59)**	**0.429**
• Ruff 2 & 7 ADS (T-s) B	45 (19–64)	45 (19–64)	46 (31–58)	0.937
*•* *Ruff 2 & 7 ADS (T-s) A*	**51.5 (30–63)**	**56.5 (30–60)**	**47 (36–63)**	**0.818**
• Ruff 2 & 7 ADA (T-s) B	48 (19–57)	46.5 (19–57)	51 (39–57)	0.394
*•* *Ruff 2 & 7 ADA (T-s) A*	**51.5 (24–59)**	**47.5 (19–55)**	**51.5 (26–55)**	**0.937**
• Ruff 2 & 7 CSS (T-s) B	43 (19–56)	44.5 (19–56)	42 (25–54)	1.000
*•* *Ruff 2 & 7 CSS (T-s) A*	**44 (24–59)**	**46 (24–59)**	**43 (30–57)**	**0.818**
• Ruff 2 & 7 CSA (T-s) BL	44 (19–60)	44 (34–59)	44.5 (19–60)	0.818
*•* *Ruff 2 & 7 CSA (T-s) A*	**45 (19–59)**	**45 (19–53)**	**45.5 (19–59)**	**0.699**
• WAIS-IV Digits forward (ss) B	10 (4–15)	11.5 (5–15)	8.5 (4–13)	0.485
*•* *WAIS-IV Digits forward (ss) A*	**9 (5–13)**	**10 (5–13)**	**9 (6–12)**	**0.699**
• WAIS-IV Digits backward (ss) B	10 (5–15)	9 (7–15)	10 (5–13)	0.818
*•* *WAIS-IV Digits backward (ss) A*	**10 (5–16)**	**9 (7–16)**	**10 (5–14)**	**1.000**
Memory				
• RAVLT immediate recall (T-s) B	42.5 (26–60)	44.5 (26–60)	40 (38–56)	0.240
*•* *RAVLT immediate recall* (*T-s) A*	**47.5 (19–67)**	**55 (19–64)**	**43 (33–67)**	**0.485**
• RAVLT delayed recall (T-s) B	46.5 (11–65)	52 (41–65)	45.5 (11–51)	0.240
*•* *RAVLT delayed recall* (*T-s) A*	**50 (39–65)**	**55.5 (39–65)**	**45 (43–62)**	**0.240**
Fatigue (raw scores)				
• MFS median (range) B	7.5 (1–22)	5.5 (4.5–21)	12.5 (1–22)	0.699
*•* *MFS, median (range) A*	**10 (2–23)**	**7.25 (2–23)**	**17 (6.5–21.5)**	**0.177**
• Fatigability median (range) B	0.5 (–10–2)*	0.5 (–3–2)	–0.5 (–10–2)	0.818
*•* *Fatigability, median (range) A*	**1 (–6–4)**	**–1 (–4–0)**	**1.5 (–6–4)**	**0.792**

BL: baseline; 8w: after 8 weeks of treatment; CWIT: Color Word Interference Test; MFS: Mental Fatigue Scale. PASAT: Paced Auditory Serial Addition Test; RAVLT: Rey Auditory Verbal Learning Test; Ruff 2 & 7 ADS: Ruff 2 & 7 Automatic Detection Speed; Ruff 2 & 7 ADA: Ruff 2 & 7 Automatic Detection Accuracy; Ruff 2 & 7 CSS: Ruff 2 & 7 Controlled Search Speed; Ruff 2 & 7 CSA: Ruff 2 & 7 Controlled Search Accuracy; WAIS: Wechsler Adult Intelligence Scale.

* one missing.

**Table V:** *D*-values of raw scores of cognitive functions and fatigue. Median and range are presented. Mann–Whitney test for comparison between groups

Factor	Total (*n* = 12)	Intervention group (*n* = 6)	Control group (*n* = 6)	*p*-value group differences
Executive functions				
• CWIT Inhibition	1.5 (–59–27)	–0.5 (–59–27)	2.5 (–16–17)	0.485
• CWIT Switching	–3.5 (–65–22)	–11.5 (–65–5)	–1 (–5–22)	0.093
Speed				
• CWIT Naming	–1.5 (–20–5)	–7.5 (–20–5)	–1 (–5–2)	0.180
• CWIT Reading	–2.5 (–13–2)	–2 (–10–2)	–3 (–13–1)	0.589
Attention				
• PASAT	4 (–4–10)	5 (2–6)	3.5 (–4–10)	0.662
• Ruff 2 & 7 ADS	15 (–16–54)	22 (–16–54)	12.5 (2–17)	0.240
• Ruff 2 & 7 ADA	–0.2 (–9.4–8.2)	0.9 (–9.4–8.2)	–0.2 (–5.0–0.9)	1.000
• Ruff 2 & 7 CSS	8.5 (–16–39)	7.5 (–8–39)	8.5 (–16–15)	1.000
• Ruff 2 & 7 CSA	–1.1 (–15.0–10.0)	–1.8 (–15.0–2.3)	–1.0 (–10.1–10.0)	0.818
• WAIS-IV Digits forward	–0.5 (–4–3)	–0.5 (–4–2)	–0.5 (–1–3)	0.818
• WAIS-IV Digits backward	0 (–6–5)	0.5 (–6–5)	0 (–1–2)	0.818
Memory				
• RAVLT immediate recall	2.5 (–14–21)	2.5 (–5–16)	3 (–14–21)	1.000
• RAVLT delayed recall	0 (–4–8)	0 (–4–4)	0 (–3–8)	0.699
Fatigue				
• MFS median	0 (–3–9)	–0.5 (–3–4.5)	1 (–2.5–9)	0.537
• Fatigability	–2 (–7–14)¤	–2 (–6–3)¤	–1 (–7–14)	0.662

BL: baseline; 8w: after 8 weeks of treatment; CWIT: Color Word Interference Test; MFS: Mental Fatigue Scale; PASAT: Paced Auditory Serial Addition Test; RAVLT: Rey Auditory Verbal Learning Test; Ruff 2 & 7 ADS: Ruff 2 & 7 Automatic Detection Speed; Ruff 2 & 7 ADA: Ruff 2 & 7 Automatic Detection Accuracy; Ruff 2 & 7 CSS: Ruff 2 & 7 Controlled Search Speed; Ruff 2 & 7 CSA: Ruff 2 & 7 Controlled Search Accuracy; Wechsler Adult Intelligence Scale.

**Table VI:** Raw scores from baseline and after 8 weeks of treatment. Median and range are presented. Wilcoxon signed rank test for comparison between baseline and follow-up

Factor	Treatment group			Control group		
	Baseline (*n* = 6)	8 weeks (*n* = 6)	*p*-value	Baseline (*n* = 6)	8 weeks (*n* = 6)	*p*-value
Executive functions						
• CWIT Inhibition	74 (42–162)	62 (40–112)	0.753	53.5 (41–81)	58 (40–98)	0.345
*• CWIT Switching*	88.5 (50–179)	74.5 (38–121)	0.075	72 (56–125)	73.5 (52–126)	0.753
Speed						
*• CWIT Naming*	42.5 (36–56)	36.5 (25–50)	0.075	34.5 (26–40)	33.5 (25–38)	0.276
*• CWIT Reading*	30 (25–38)	29 (22–32)	0.248	28.5 (22–40)	24.5 (21–30)	0.058
Attention						
• PASAT	41 (24–55)	53 (29–60)*	0.042	39 (17–49)	42.5 (13–59)	0.176
• Ruff 2&7 ADS	115 (38–166)	138.5 (61–175)	0.075	120 (87–155)	126.5 (103–169)	0.027
• Ruff 2&7 ADA	95.5 (73–100)	96 (81–99)	0.917	97.5 (92–100)	98 (87–99)	0.463
• Ruff 2&7 CSS	104.5 (42–114)	105 (53–153)	0.173	102.5 (73–129)	104 (85–137)	0.344
• Ruff 2&7 CSA	90.5 (86–98)	92 (71–98)	0.249	91 (77–99)	90 (74–98)	0.463
• WAIS-IV Digit forward -score	9.5 (6–12)	8.5 (6–11)	0.680	8 (5–11)	8 (7–10)	1.000
• WAIS-IV Digit span forward	6 (4–7)	6.5 (5–7)	0.317	6 (4–7)	6 (5–6)	1.000
• WAIS-IV Digit backward – score	7 (6–12)	7.5 (6–12)	0.786	8 (5–10)	8 (5–11)	0.655
• WAIS-IV Digit span backward	4 (3–7)	4.5 (3–7)	0.713	5 (3–6)	5 (3–6)	0.564
Memory						
• RAVLT immediate recall	40.5 (28–52)	48 (23–63)	0.293	39.5 (29–54)	42.5 (34–62)	0.416
• RAVLT delayed recall	10 (6–12)	10.5 (6–12)	0.786	8 (5–11)	8 (8–13)	1.000
Fatigue						
• MFS	5.5 (4.5–21)	7.25 (2–23)	0.893	12.5 (1–22)	17 (6.5–21.5)	0.498
• Fatigability	0.5 (–3–2)	–1 (–4–0)*	0.223	–0.5 (–10–2)	1.5 (–6–4)	0.715

BL: baseline; 8w: after 8 weeks of treatment; CWIT: Color Word Interference Test; MFS: Mental Fatigue Scale; PASAT: Paced Auditory Serial Addition Test; RAVLT: Rey Auditory Verbal Learning Test; Ruff 2 & 7 ADS: Ruff 2 & 7 Automatic Detection Speed; Ruff 2 & 7 ADA: Ruff 2 & 7 Automatic Detection Accuracy; Ruff 2 & 7 CSS: Ruff 2 & 7 Controlled Search Speed; Ruff 2 & 7 CSA: Ruff 2 & 7 Controlled Search Accuracy; Wechsler Adult Intelligence Scale.

* One missing.

### Results from fMRI investigation

**Fig. 2:**
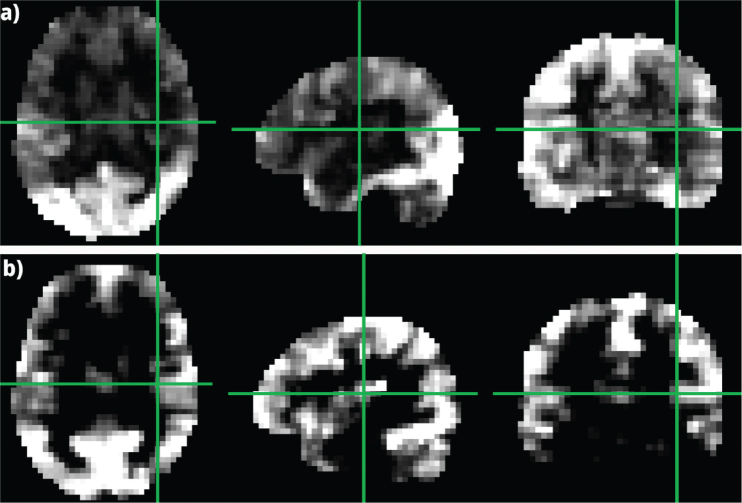
Comparison of FC metrics: (A) cross-sectional display of CSI metric for a stroke patient (subject #4 in the intervention group) with a lesion in the right temporal lobe (as indicated by the crossing green lines) and healthy subjects, (B) cross-sectional display of CSI metric for a healthy control at the same anatomic position.

*Functional connectivity.* Fig. 2 provides compelling evidence that cerebral ischaemia lesions significantly alter FC metrics. This disruption extends beyond the immediate lesion site, impacting broader brain networks. By comparing FC in the affected hemisphere with the ipsilateral side of healthy subjects, we observed a marked depletion of FC associated with the lesion.

The PCA analysis at baseline revealed that the first 2 components captured over 85% of the total variance in the data. Notably, there was no clear separation between intervention and control groups, suggesting that clinical data for both groups were well mixed. The executive functions were the main contributors to PCA1, including CWIT subtests Inhibition and Switching performances, while PCA2 was primarily determined by attention including Ruff 2 & 7 Automatic Detection Speed (ADS) and Ruff 2 & 7 Controlled Search Speed (CSS). The intervention was not associated with any statistically significant changes in these components. This is consistent with the results of the ANOVA test of the FC metrics. Neither CSI nor CDI showed any significant difference between the intervention and control subjects at the assessments at baseline and after 8 weeks of intervention.

**Fig. 3:**
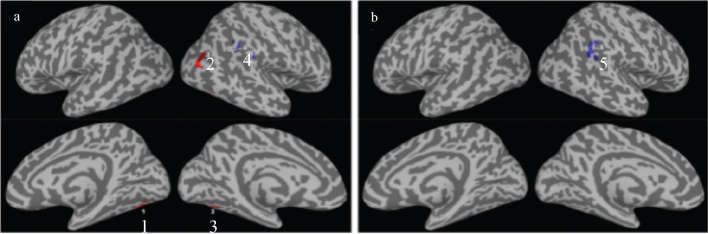
Summary of linear regression results for the FC metric CSI vs the top 2 PCA components of the clinical data of all participants. Brain regions with significant correlations at voxel-wise *p*<0.01 and cluster size >18 are depicted in colours, for (A) PCA1 and (B) PCA2 respectively. The annotated numerical numbers are the numbering of the ROIs defined in Table VII.

Linear regression analysis (Table VII, Fig. 3) highlights the relationship between FC metrics and PCA components. Specifically, FC in the bilateral fusiform gyrus was positively correlated (red colour) with PCA1. In contrast, FC in the right angular gyrus and inferior parietal lobule (IPL) were negatively correlated (blue colour) with both PCA1 and PCA2. It is important to note that the precise anatomical locations associated with these correlations differ slightly, with the region linked to PCA2 being more posterior and inferior.

**Table VII:** Summary of linear regression results for the CSI metric vs the top 2 PCA components

ROI	PCA	VOL	CM_RL_	CM_AP_	CM_IS_	*r*	Overlap with Atlas
1	1	21	38.5	61.9	–19.2	0.71	64.5% L-Fusiform Gyrus, 32.1% L-Cerebellum
2	1	20	–38.3	81.8	22.8	0.63	98.4% R-Middle Occipital Gyrus
3	1	18	–40.3	69	–19.6	0.71	48.8% R-Fusiform Gyrus, 50.3% R-Cerebellum
4	1	18	–54.2	48.5	37.3	–0.59	25.6% R-Angular Gyrus, 49.5% R-IPL, 24.9% R-SupraMarginal Gyrus
5	2	27	–55.6	54.4	30.1	–0.76	71.8% R-Angular Gyrus, 9.9% R-IPL, 8.7% R-MTG, 8% r-STG, 1.2% R-Supra Marginal

## DISCUSSION

This pilot RCT aimed to explore the effects of AE on memory, attention, working memory, and fatigue in patients with stroke or TBI. Preliminary findings showed no significant benefit of AE on any of the cognitive functions or on fatigue in comparison with the controls. However, when measuring intragroup differences, we found significant improvement on the PASAT test in the AE group but not in the control group. This is a test measuring auditive processing speed and working memory. There were also tendencies for improvements (*p*<0.08) in the AE group on the CWIT subtests Naming and Switching – tests that also demand a combination of processing speed and executive functioning. This was not seen in the control group. On the other hand, the control group performed significantly better on the Ruff 2 & 7 test of automatic detection speed after 8 weeks of treatment compared with baseline and had a tendency for improvement on the CWIT reading condition, which also is a test of simple processing capacity. To summarize, preliminary findings showed that the AE group improved more on tests demanding higher order cognitive functions while the control group improved more on basic tasks of processing speed. fMRI-based assessments revealed no preliminary signs of significant differences in cerebral functional connectivity between the groups following rehabilitation.

Previous studies on the role of aerobic exercise in cognition have shown inconclusive results. A systematic review on physical exercise and cognition after stroke or TBI found that exercise lasting 8 weeks or more improved cognition. Specifically, AE was shown to have a positive impact in several quality studies. The review also suggested that combining AE with resistance training (RT) could be particularly effective for enhancing cognition in this population (37). A systematic review focusing only on stroke patients found that there was a significant positive effect of physical activity on cognition and thus supported the use of physical training as a treatment strategy to promote cognitive recovery after stroke (38). On the other hand, a randomized controlled trial with 47 participants investigated the impact of either 6 months of AE or low-intensity non-aerobic exercises on cognitive function after stroke and found no positive benefits of exercise (39). Studies on TBI patients and the role of physical exercise in cognitive recovery are fewer than those for stroke, and firm conclusions regarding the effect are lacking due to methodological shortcomings (40).

### Measurements of neuroplasticity

A systematic review of the impact of AE on neuroplasticity in stroke patients, measured through fMRI, concluded that AE could potentially modify the neural network. Moreover, moderate- to high-intensity exercise seemed to create a higher neuroplastic response. What intensity was needed was not concluded. Notably, one key finding was that light-intensity exercise did not result in changes in cortical excitability and cognitive stimuli after an exercise session seemed to provide additional neuroplastic benefit (41). Another systematic review concluded that high-intensity interval training improves post-stroke recovery by increasing neuroplasticity markers such as BDNF (17). In our study, patients exercised on 60–80% of HR-max, i.e., within the range of moderate- to high-intensity exercise, which would potentially have resulted in neuroplastic changes detected by fMRI. We did not observe such changes. However, our study was underpowered, and the lack of effects could be due to the small number of participants. Also, we did not include a passive control group. Rather our controls were subject to other rehabilitation intervention that could have given neuroplastic changes.

### Methodological aspects and limitations

This is an exploratory pilot RCT involving a selected group of patients with ABI. Although the study has clear limitations, it can underscore the importance of carefully considering methodological factors, both in this study and in earlier work when evaluating the effects of AE on cognition and/or fatigue.

*Dose.* There is an ongoing debate regarding the optimal type, intensity, duration, and frequency of physical exercise to boost brain plasticity and cognitive function (42, 43). While some studies demonstrate a clear dose–response relationship (44), there are currently no established protocols outlining the ideal dose of AE post-stroke or TBI to maximize cognitive or fatigue-related benefits. For instance, the exercise regimen used in this study, 60–80% of HR-max for 30 min, 3–4 times per week raises the question: Is this sufficient to promote cognitive function? Research indicates that patients with mild cognitive impairment (MCI) show significant improvements with an exercise dose of ≥ 3 sessions per week, vigorous intensity, and 30–60 min per session (45). Although direct comparisons between these groups may not be valid, it underscores the need to question whether the exercise dose in this study was optimal.

An increase in peak oxygen uptake might influence cognition (46) or fatigue. This study focused on aerobic exercise as a way of possibly influencing cognition or fatigue, not taking into consideration the level of endurance before and after rehabilitation. Further studies might consider comparing the possible improvements of endurance with the outcome of NP tests or fatigue.

This pilot RCT used AE as the intervention. Comparisons with previous research are challenging due to inconsistent use of terms such as physical activity, exercise, and training. By clearly defining our metho-dology, this pilot RCT aims to support systematic reviews focused specifically on AE as a means to improve cognition and/or fatigue, and to contribute to the development of evidence-based exercise prescriptions.

*Priming.* Priming can be described by behavioural changes generated by preceding stimuli (47, 48). The reorganization and creation of new networks is modulated by stimuli received (49). If increased levels of BDNF and other factors influencing neuroplasticity are to have an impact on cognition after aerobic exercise, the aerobic training should presumably be followed by cognitive training or stimuli for optimal results. Although studies have shown an increase in BDNF levels after aerobic exercise, it is not known how long the levels remain elevated. This window of opportunity is of importance, as the ability to improve cognition should be greatest when the levels are elevated. In this pilot RCT, all patients participated in an individualized rehabilitation programme. However, the activities following the aerobic exercise were not documented. All patients had a weekly schedule for their rehabilitation based on an individualized rehabilitation plan. The schedule could change from day to day and week to week. Thus, there was no way of knowing or scheduling what happened after the intervention in the AE group, which is an obvious limitation.

*Patient selection.* In our patient selection process, we excluded individuals with moderate depression and/or anxiety, as these conditions are known to impact both fatigue (50) and cognition. Additionally, AE has been shown to reduce symptoms of depression. Including patients with depression or anxiety would have made it difficult to distinguish whether any observed effects were due to improved cognitive function or decreased anxiety and depression. These strict exclusion criteria are disputable and excluded many patients from participating in this pilot RCT. HADS primarily measures the probability of depression. Many patients with ABI score high on items in the HADS, not due to depression but due to other consequences of their injury.

Despite the extended study period, rehabilitation guidelines for the included patient groups remained consistent. While minor variations in implementation may have occurred, the core principles did not change. This consistency was crucial to ensure comparability between groups throughout the study.

A more notable limitation of this pilot RCT is the absence of detailed documentation regarding the number of patients excluded and the specific reasons for their exclusion during the selection process.

*Statistical power.* This is an RCT pilot study with only 12 participants with either stroke or TBI. It was initially designed as a randomized controlled study with a neuropsychological test’s primary outcome measure. The plans were accordingly to enrol 16 patients in each group. Not having access to an MRI evaluation during a couple of years limited the numbers of patients being recruited and then the COVID-19 pandemic became a factor, making recruitment impossible due to hospital restrictions. It was then decided to stop the study but report our findings and experience. The limited numbers of patients obviously affect the possibility of reaching a significant conclusion and results should therefore be looked at as preliminary.

*fMRI.* The initial idea was to explore whether we could find any associations between changes/improvements in cognitive function and changes/improvements in connectivity, i.e., structural-functional correlations. Although preliminary findings did not observe any significant between-group differences after intervention, our results at baseline indicate that the groups were well matched for clinical/demographic data. Furthermore, and within groups, we observed correlations between neuropsychological function and FC metrics in brain regions associated with higher cognitive functions. The bilateral fusiform gyrus was positively correlated with PCA1. Preliminary results from the executive test CWIT (inhibition and switching) were significantly correlated to PCA1. This aligns well with the role of the fusiform gyrus as it has been understood to be involved in the processing of high-order visual information (51) and the CWT task demands executive processing of visual information (52). In contrast, FC in the right angular gyrus was negatively correlated with both PCA1 and PCA2. The function of the angular gyrus has been proposed to have a causal role in, e.g., semantic processing, word and number processing, attention, and visual search (53). As PCA2 was significantly correlated with the preliminary results from the attention test Ruff 2 & 7 (ADS and CSS) this is also in line with expected associations between test results and functional connectivity in the brain. These preliminary findings suggest a potential link between these cognitive functions and the underlying neural networks and encourage the use of sensitive neuropsychological tests in a future efficacy study.

### Physiological responses stroke vs TBI

It is important to point out that the 2 patient groups may exhibit different physiological responses to AE. Stroke typically results in focal brain lesions that can lead to asymmetric autonomic dysfunction, diminished baroreflex sensitivity, and consequently an attenuated heart rate increase during exercise (54). In contrast, TBI often produces diffuse autonomic dysregulation, characterized by sympathetic hyperactivity and reduced heart-rate variability (55). The 2 patient groups might therefore have reacted differently to the AE. Further studies might therefore want to separate the 2 groups.

### Conclusion

The question of whether aerobic exercise promotes cognition and fatigue after brain injury remains unanswered. This pilot RCT and its preliminary findings indicate greater improvements in executive processing speed in the aerobic exercise group, while more automatized attention speed improved more in the control group. The experiences from this study might facilitate the design of future studies on this intriguing topic, for instance multicentre RCTs to maximize the number of possible participants.
